# Maternal obesity and offspring cardiovascular remodelling — the effect of preconception and antenatal lifestyle interventions: a systematic review

**DOI:** 10.1038/s41366-024-01536-0

**Published:** 2024-06-19

**Authors:** Samuel J. Burden, Rahaf Alshehri, Pablo Lamata, Lucilla Poston, Paul D. Taylor

**Affiliations:** 1 Department of Women and Children’s Health, School of Life Course & Population Sciences, King’s College London, London, UK; 2 Cardiovascular Medicine and Science Research, School of Cardiovascular and Metabolic Medicine & Sciences, King’s College London, London, UK; 3 Biomedical Engineering, School of Biomedical Engineering & Imaging Sciences, King’s College London, London, UK

**Keywords:** Cardiovascular diseases, Risk factors, Cardiovascular diseases, Anatomy, Preclinical research

## Abstract

**Background:**

Preconception or antenatal lifestyle interventions in women with obesity may prevent adverse cardiovascular outcomes in the child, including cardiac remodelling. We undertook a systematic review of the existing data to examine the impact of randomised controlled trials of lifestyle interventions in pregnant women with obesity on offspring cardiac remodelling and related parameters of cardiovascular health.

**Methods:**

This review was registered with PROSPERO (CRD42023454762) and aligns with PRISMA guidelines. PubMed, Embase, and previous reviews were systematically searched. Follow-up studies from randomised trials of lifestyle interventions in pregnant women with obesity, which included offspring cardiac remodelling or related cardiovascular parameters as outcome measures, were included based on pre-defined inclusion criteria.

**Results:**

Eight studies from five randomised controlled trials were included after screening 3252 articles. Interventions included antenatal exercise (*n* = 2), diet and physical activity (*n* = 2), and preconception diet and physical activity (*n* = 1). Children were <2-months to 3–7-years-old, with sample sizes ranging between *n* = 18–404. Reduced cardiac remodelling, with reduced interventricular septal wall thickness, was consistently reported. Some studies identified improved systolic and diastolic function and a reduced resting heart rate. Risk of bias analyses rated all studies as ‘fair’ (some risk of bias). A high loss-to-follow-up was a common limitation.

**Conclusion:**

Although there is some evidence to suggest that lifestyle interventions in women with obesity may limit offspring cardiac remodelling, further high-quality longitudinal studies with larger sample sizes are required to confirm these observations and to determine whether these changes persist to adulthood.

**Figure:**
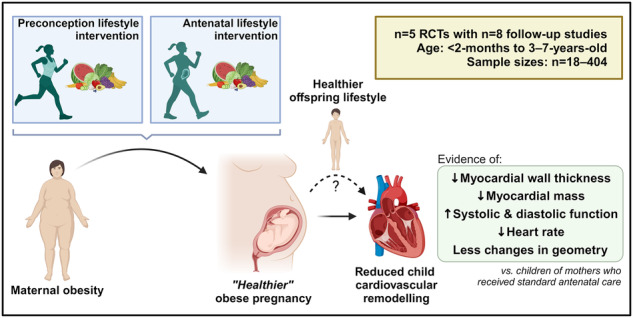
Child offspring cardiovascular health benefits of preconception and antenatal lifestyle interventions in women with obesity.

## Introduction

Obesity rates during pregnancy are increasing globally, with more than 50% of women who attend antenatal clinics in England and Wales being classified as having overweight (28.5%) or obesity (22.7%) [1]. The Developmental Origins of Health and Disease (DOHaD) concept suggests that non-communicable diseases, such as cardiovascular disease may, at least in part, have origins in adverse environmental exposures during preconception, in utero, and in early infancy (“the first thousand days” hypothesis) [2, 3]. Numerous studies in rodent models of maternal obesity have demonstrated cardiac structural changes, cardiovascular dysfunction, and reduced cardiometabolic health in the offspring [4, 5], with reported prevention through exercise interventions in the pregnant dam [6, 7]. Relevance to human health is derived from population based observational studies which have reported that children of mothers with obesity are predisposed to future adverse cardiovascular health outcomes, with increased risk of premature cardiovascular and all-cause mortality [8, 9].

Recent reviews have summarised the impact of maternal obesity on offspring cardiovascular health and concluded, from observational human studies, that maternal body mass index (BMI) is associated with childhood cardiovascular remodelling [4, 10, 11]. However, it was identified that this relationship may also be compounded by childhood BMI. Together with the problem of residual confounding in observational studies, the complex interplay between in utero origins of cardiovascular health, a shared postnatal lifestyle environment, and genetic predisposition, causality is difficult to establish. Since these reviews were published [4, 10, 11], further observational studies have reported similar relationships between maternal obesity and offspring cardiovascular health [12–14].

Most recently, several randomised controlled trials (RCTs) of lifestyle interventions in women with obesity have been undertaken [15–22], with longitudinal follow-up of offspring cardiovascular health. Given the call for primary prevention for obesity-related cardiovascular disease [23, 24], the intention of this systematic review was to synthesise the data from these RCTs. We have examined the impact of preconception and antenatal lifestyle interventions in women with obesity on offspring cardiac remodelling (cardiac structure and function) and related cardiovascular outcomes (blood pressure, heart rate, and arterial thickening/stiffness), and have highlighted knowledge gaps that require further study.

## Methods

The protocol for this systematic review was registered with PROSPERO International Prospective Register of Systematic Reviews (https://www.crd.york.ac.uk/prospero/; Identifier: CRD42023454762). Reporting was in accordance with the 2020 Preferred Reporting Items for Systematic Reviews and Meta-Analyses (PRISMA) guidelines [25]. The PRISMA checklist is provided in Supplementary Table S1.

### Eligibility criteria

We included follow-up studies from RCTs of children born to mothers with overweight (BMI ≥ 25 kg/m^2^) or obesity (BMI ≥ 30 kg/m^2^) who had participated in a preconception or antenatal lifestyle RCT (diet and/or physical activity). Studies were included if the child was assessed for cardiac structure and/or function or a related cardiovascular outcome (blood pressure, heart rate, or arterial thickening/stiffness) as a child or adult. RCTs that focussed on other comorbidities typically associated with obesity, such as gestational diabetes or hypertensive disorders of pregnancy, that also investigated the impact of maternal overweight/obesity on childhood cardiovascular outcomes were included.

All publications identified by the literature search were independently reviewed by two authors (SJB and RA), with any discrepancies resolved by inclusion of a third author (PDT). Inclusion was limited to full-text articles reported in English and published in peer-reviewed journals. Full inclusion and exclusion criteria are provided in Table 1. Manuscript screening was managed using Rayyan (http://rayyan.qcri.org/) [26].

**Table 1:** Inclusion and exclusion criteria.

	Inclusion	Exclusion
Exposure	• Preconception or antenatal lifestyle (diet and/or physical activity) randomised control trial (RCT) in women with overweight or obesity. • Preconception or antenatal lifestyle RCTs focussing on comorbidities associated with obesity, such as gestational diabetes mellitus or hypertension, that also investigated the impact of maternal overweight or obesity.	• RCTs that solely focussed on women with normal weight. • RCTs utilising nutritional supplements (e.g. calcium supplementation) or drug trials.
Outcome	• Cardiac structure. • Cardiac function (systolic and diastolic function). • Heart rate and heart rate variability. • Arterial stiffness and arterial thickness (e.g. pulse-wave velocity). • Blood pressure.	• Cardiometabolic health (e.g. blood lipid concentrations). • Focus on other aspects of offspring health (e.g. adiposity). • Focus on maternal health. • Fetal cardiovascular remodelling.
Time frame	• Offspring of any age (paediatric or adult).	
Study design	• Follow-up studies of RCTs. • Original research study. • Quantitative studies. • Human studies.	• Reviews and meta-analyses. • Case reports. • Opinion papers. • Animal studies.
Availability	• Full-text available. • Articles reported in English and published in peer-reviewed journals.	• Published in grey literature. • Conference or meeting abstracts. • Not written in English.

### Search strategy

Search terms were devised by one author (SJB) and checked for completeness and correctness by two others (PDT and PL). Common terms and key words such as obesity, maternal, cardiovascular, cardiac, children/offspring, trial, and follow-up were combined in search hedges and were applied in PubMed.gov (1958 to present) and Embase (1974 to present). The full search strategy is reported in Supplement Tables S2 and S3. The literature search was completed on the 31st of August 2023. Reference lists of pertinent review articles [4, 10, 11] were also screened for any studies that were not captured by the database search, although this yielded no further studies. Data from included studies were populated into predefined tables by one author (SJB).

The protocol and primary outcome paper for each RCT were also obtained from the reference lists of the included follow-up studies, or by searching for the trial registry number online, to assist with the risk of bias assessment, to provide a summary of the maternal intervention, and to describe any limitations in the RCT design.

### Risk of bias assessment

The Quality Assessment Tool for Observational Cohort and Cross-Sectional Studies by the National Heart, Lung and Blood Institute (NHLBI) was used to assess quality and risk of bias [27]. Question 3 of this tool was replaced with question 7 from the NHLBI Quality Assessment of Case-Control Studies, as this better reflected sampling from an established trial cohort. Any recruitment and/or randomisation bias in the original RCTs were assessed by questions 2.4, 5.4, and 5.5 from the ROBINS-I tool [28] and domain 1 of the RoB 2 tool [29], respectively. Scores of “good” (least risk of bias), “fair” (susceptible to some bias) and “poor” (significant risk of bias) were given to each study based on study design and implementation. Further explanation of how these tools assess risk of bias are provided online or in their dedicated publication [27–29].

### Reporting

Results from individual studies were reported in outcome specific tables. Significant results were also reported in-text as mean differences (MD) and 95% confidence intervals (CIs) of the intervention versus non-intervention arm. If the MD and 95% CIs were not reported in the individual studies, then the MD was calculated as MD = intervention mean − non-intervention mean. If *p*-values were reported instead of 95% CIs, then approximate 95% CIs were estimated using MD ± 1.96*standard error (SE). Approximate SEs were calculated as SE = MD/z-score (*z*-scores: two-tailed *p*-values converted to *z*-scores). If *p*-values were reported as *p* < 0.001, then a conservative value of *p* = 0.001 was used. Due to the exploratory nature of the included studies, results with a *p*-value of <0.10 were also highlighted in-text but were labelled as non-significant.

## Results

**Fig. 1:**
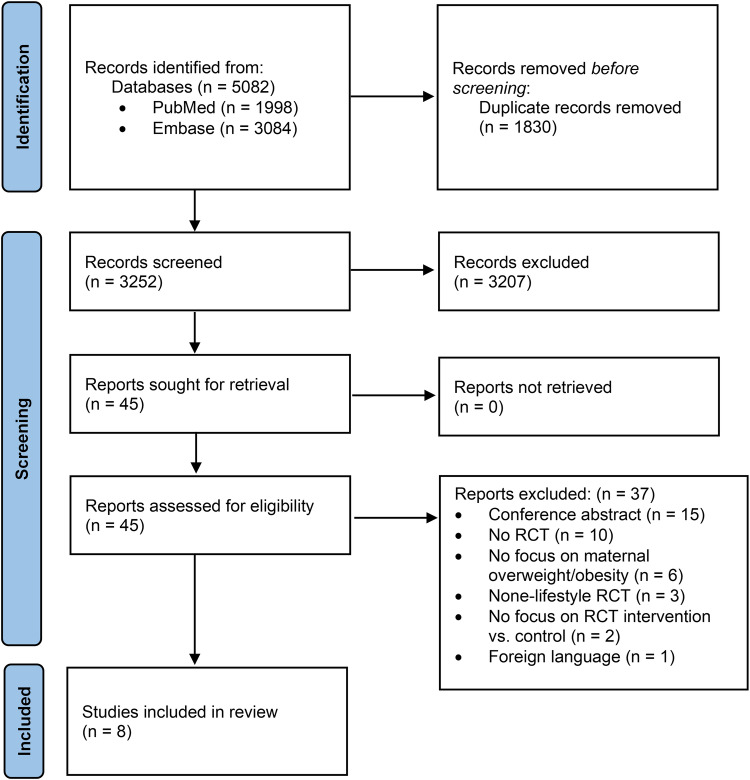
PRISMA flow chart. Flow chart of study identification, screening, and reasons for inclusion and exclusion.

Searches identified 5082 studies. After duplicate removal, 3252 were screened, and 3207 were excluded, leaving 45 reports to be assessed (Fig. 1). A total of eight studies were included in the systematic review.

### Study characteristics

A summary of each RCT, the baseline maternal characteristics, and offspring characteristics at follow-up is provided in Table 2.

**Table 2:** Study characteristics.

Study	Intervention summary	Baseline maternal characteristics			Follow-up offspring characteristics			Cardiovascular assessments	Quality score
		Intervention	Non-intervention	Normal weight comparator group	Intervention	Non-intervention	Normal weight comparator group		
ENHANCED May, 2023 [15]	Setting: Single centre, United States of America Sample size: *n* = 140 randomised Start: 13–16 weeks GA; Duration: 24+ weeks Protocol: Antenatal exercise. In-person, 3× per week, 50 min moderate-intensity aerobic exercise (intervention group) OR 50-minute low-intensity stretching and breathing techniques (“attention control” group). Adherence to intervention: nr Maternal outcome: Intervention group did more exercise per week than non-intervention group.	BMI: 25.7 ± 5.5* (Pre-pregnancy, between 18.5–39.0 kg/m^2^) *n* = 70 randomised *n* = 44 completed Age: 30.1 ± 3.2 years (between 18–40 years, pre-pregnancy age) Ethnicity: nr	BMI: 25.0 ± 4.7* (Pre-pregnancy, between 18.5–39.0 kg/m^2^) *n* = 70 randomised *n* = 55 completed Age: 30.5 ± 5.2 years (between 18–40 years, pre-pregnancy age) Ethnicity: nr	None	*n* = 31 (44% of randomised) Follow-up period: 4–5 weeks postnatal Age: nr Sex (m/f): 19/12 Maternal ethnicity: nr	*n* = 25 (36% of randomised) Follow-up period: 4–5 weeks postnatal Age: nr Sex (m/f): 11/13 Maternal ethnicity: nr	None	Echocardiography, heart rate (heart rate measured during echo [likely ECG])	Fair
ETIP Nyrnes, 2018 [34]	Setting: Single centre, Norway Sample size: *n* = 91 randomised Start: 12–18 weeks GA; Duration: Until delivery Protocol: Antenatal exercise. In-person, 3x per week, 60 min exercise (35 min endurance + 25 min resistance training) & 1× per week home, 50 min exercise (35 min endurance + 15 min strength exercise) OR standard care. Adherence to intervention: 50% Maternal outcome: Increased exercise during late pregnancy in intervention group. No difference between groups in GWG or other anthropometric measures. Reduced prevalence of GDM and lower systolic blood pressure in intervention group.	BMI: 33.9 ± 3.8 (Pre-pregnancy, ≥28 kg/m^2^) *n* = 46 randomised *n* = 38 completed Age: 31.3 ± 3.8 years (≥18 years, pre-pregnancy age) Ethnicity: nr	BMI: 35.1 ± 4.6 (Pre-pregnancy, ≥28 kg/m^2^) *n* = 45 randomised *n* = 36 completed Age: 31.1 ± 4.7 years (≥18 years, pre-pregnancy age) Ethnicity: nr	Normal weight BMI: 21.0 ± 2.3 (Pre-pregnancy, between 18.5–25.0 kg/m^2^) *n* = 20 recruited (1–3 days follow-up *n* = 18; 6–8 weeks follow-up *n* = 20) Age: 31.2 ± 4.1 years (≥18 years) Ethnicity: nr	*n* = 23–26 (50–57% of randomised) Follow-up period: 1–3 days & 6–8 weeks postnatal Age 1st visit: 2 (1, 3) days Age 2nd visit: 8.6 (7.8, 9.6) weeks Sex: nr Maternal ethnicity: nr	*n* = 27 (60% of randomised) Follow-up period: 1–3 days & 6–8 weeks postnatal Age 1st visit: 2 (1,3) days Age 2nd visit: 7.7 (7.1, 9.0) weeks Sex: nr Maternal ethnicity: nr	Offspring of normal-weight mothers *n* = 20 Follow-up period: 1–3 days & 6–8 weeks postnatal Age 1st visit: 3 (2, 3) days Age 2nd visit: 8.4 (7.1, 12.1) weeks Sex: nr Maternal ethnicity: nr	Echocardiography, blood pressure, and heart rate (heart rate measured by ECG)	Fair
Lifestyle Mintjens, 2021 [33]	Setting: Multicentre (23 sites), Netherlands Sample size: *n* = 577 randomised Start: After 12 months attempting to conceive naturally; Duration: 6 months + infertility treatment (24 months total) Protocol: Preconception (subfertile women) physical activity and diet. Six outpatient appointments and four telephone consultations. Goal of 5–10% loss of body weight. Reduce energy intake by 600 kcal (minimum 1200 kcal per day), target 10,000 steps per day, and 2–3 times per week of ≥30 min moderate-intensity exercise, preceding 18 months infertility treatment OR immediate 24 months of infertility treatment. Adherence to intervention: nr Maternal outcome: 37.7% of intervention group lost ≥5% of their original body weight; 10.5% of non-intervention group lost ≥5% over the first 6 months. 43.0% of those who completed intervention lost ≥5% of their original bodyweight.	BMI: 36.0 (33.4–38.2) (≥29 kg/m^2^) *n* = 290 randomised *n* = 222 completed Age: 29.7 ± 4.5 (18–39) years Ethnicity (white ethnic origin): 256 (88.6%)	BMI: 36.0 (33.5–38.2) (≥29 kg/m^2^) *n* = 287 randomised *n* = 284 completed Age: 29.8 ± 4.6 (18–39) years Ethnicity (white ethnic origin): 246 (86.3%)	None	*n* = 17 (12% of 145 eligible births) Follow-up period: 3–6 years old Age: 4.6 ± 1.0 years (both arms combined) Sex: 22 male (48%) (both arms combined) Maternal ethnicity (Caucasian): 16 (94%)	*n* = 29 (18% of 160 eligible births) Follow-up period: 3–6 years old Age: 4.6 ± 1.0 years (both arms combined) Sex: 22 male (48%) (both arms combined) Maternal ethnicity (Caucasian): 28 (97%)	None	Blood pressure, pulse-wave velocity	Fair
Lifestyle den Harink, 2022 [31]	*Same as Mintjens 2021*	*Same as Mintjens 2021*	*Same as Mintjens 2021*	None	*n* = 24 (8% of 319 eligible children [both arms combined]) Follow-up period: nr Age: 6.6 ± 1.2 Sex (m/f): 11/13 Maternal ethnicity: nr	*n* = 36 (11% of 319 eligible children [both arms combined]) Follow-up period: nr Age: 6.5 ± 1.0 Sex (m/f): 18/18 Maternal ethnicity: nr	None	Echocardiography, heart rate (mean heart rate during echo [likely ECG]), and carotid ultrasound	Fair
Lifestyle den Harink, 2023 [32]	*Same as Mintjens 2021*	*Same as Mintjens 2021*	*Same as Mintjens 2021*	None	*n* = 29 (9% of 319 eligible children [both arms combined]) Follow-up period: ≥6 years old Age: 7.2 ± 0.7 Sex (m/f): 15/14 Maternal ethnicity: nr	*n* = 20 (6% of 319 eligible children [both arms combined]) Follow-up period: ≥6 years old Age: 7.1 ± 1.0 Sex (m/f): 10/10 Maternal ethnicity: nr	None	Cardiac MRI, heart rate (mean heart rate during MRI [ECG])	Fair
LiP Tanvig, 2015 [35]	Setting: Multicentre (2 sites), Denmark Sample size: *n* = 360 randomised Start: 15 weeks GA; Duration: Until delivery Protocol: Antenatal physical activity and diet. 4x dietary counselling sessions. Encouragement to do 30–60 min moderate-intensity physical activity per day. Fitness centre membership, one hour per week with physiotherapist (aerobic [low-step], light weights and elastic bands, and balance exercises), preceding 4–6 group physiotherapy sessions. Goal of limiting gestational weight gain to 5 kg. OR standard care. Adherence to intervention: 92% completed all 4 sessions; 98% completed ≥3 sessions. 56% attended at least half of the aerobic classes. 78% undertook leisure time sport, compared to 65% in non-intervention group. Maternal outcome: Intervention group had less GWG (1.6 kg) than non-intervention group.	BMI: 33.4 (31.7–36.5) (Pre-pregnancy, between 30–45 kg/m^2^) *n* = 180 randomised *n* = 150 completed Age: 29 (27–32) years (18–40 years, pre-pregnancy age) Ethnicity (Caucasians): 150 (100%)	BMI: 33.3 (31.7–36.9) (Pre-pregnancy, between 30–45 kg/m^2^) *n* = 180 randomised *n* = 154 completed Age: 29 (26–31) years (18–40 years, pre-pregnancy age) Ethnicity (Caucasians): 154 (100%)	Normal weight BMI: 22.1 (20.7–23.4) (Pre-pregnancy, between 18.5–24.9 kg/m2) *n* = 97 Age: 30.2 (28.0–33.1) years (18–40 years, age at delivery) Ethnicity (Caucasians): 97 (100%)	*n* = 77 (43% of randomised) Follow-up period: 2.5–3.2 years old Age: 2.8 (2.8–2.9) years Sex (m/f): 39/38 Maternal ethnicity (Caucasians): *assumed 100%*	*n* = 73 (41% of randomised) Follow-up period: 2.5–3.2 years old Age: 2.8 (2.8–2.9) years Sex (m/f): 41/32 Maternal ethnicity (Caucasians): *assumed 100%*	Offspring of normal-weight mothers *n* = 97 Follow-up period: 2.5–3.2 years old Age: 2.8 (2.8–2.9) years Sex (m/f): 50/47 Maternal ethnicity (Caucasians): *assumed 100%*	Blood pressure	Fair
UPBEAT Dalrymple, 2021 [30]	Setting: Multicentre (8 sites), United Kingdom Sample size: *n* = 1555 randomised Start: Within one week of randomisation (15–18 weeks GA); Duration: Until delivery Protocol: Antenatal physical activity and diet. 9 × 1–1.5 h sessions with health trainer, in-person or telephone/email. Handbook for diet and DVD for physical activity regime. Goal to reduce glycaemic load and incrementally increase daily step count tailored to participants lifestyle OR standard care. Adherence to intervention: on average women participated in 7/8 (plus initial visit). 30% of women attended one in-person session, and 46% attended <4. Of all sessions, 10% received 1 session and 17% had <4. Maternal outcome: There was no difference in the primary outcomes (GDM or LGA). The intervention group spent more time walking during pregnancy, had a reduced glycaemic index, and had less GWG (0.6 kg) when compared to non-intervention group.	BMI: 36.3 ± 5.0 (antenatal 15–18 weeks, ≥30 kg/m^2^) *n* = 783 randomised *n* = 629 primary maternal outcomes *n* = 761 primary neonatal outcomes Age: 30.5 ± 5.5 years (>16 years, age at antenatal visit 15–18 weeks) Ethnicity (white ethnic origin): 490 (63%)	BMI: 36.3 ± 4.6 (antenatal 15–18 weeks, ≥30 kg/m^2^) *n* = 772 *n* = 651 primary maternal outcomes *n* = 751 primary neonatal outcomes Age: 30.4 ± 5.6 years (>16 years, age at antenatal visit 15–18 weeks) Ethnicity (white ethnic origin): 483 (63%)	None	*n* = 250 (33% of 765 with known birthweight) Follow-up period: 3 years old Age: 3.5 ± 0.3 years Sex: nr Maternal ethnicity (white ethnic origin): 173 (69%)	*n* = 264 (35% of 757 with known birthweight) Follow-up period: 3 years old Age: 3.5 ± 0.3 years Sex: nr Maternal ethnicity (white ethnic origin): 176 (67%)	None	Blood pressure and heart rate (heart rate measured using blood pressure machine)	Fair
UPBEAT Taylor, 2022 [14]	*Same as Dalrymple 2021*	*Same as Dalrymple 2021*	*Same as Dalrymple 2021*	Normal-weight BMI: 22.7 (20.9–23.6) (antenatal 15–18 weeks, 20–25 kg/m^2^) *n* = 52 Age: 32.6 ± 4.4 years (>16 years, age at delivery) Ethnicity (white ethnic origin): 37 (71%)	*n* = 31 (4% of 765 with known birthweight) Follow-up period: 3 years old Age: 3.7 ± 0.2 years Sex (m/f): 16/15 Maternal ethnicity (white ethnic origin): 16 (41%)	*n* = 39 (5% of 757 with known birthweight) Follow-up period: 3 years old Age: 3.7 ± 0.2 years Sex (m/f): 17/22 Maternal ethnicity (white ethnic origin): 13 (42%)	Offspring of normal-weight mothers *n* = 52 Follow-up period: 3 years old Age: 3.9 ± 0.1 years Sex (m/f): 20/32 Maternal ethnicity (white ethnic origin): 37 (71%)	Echocardiography, carotid ultrasound, pulse-wave velocity, blood pressure, heart rate and heart rate variability (measured by ECG)	Fair

*BMI* body mass index, *ECG* electrocardiogram, *GA* gestational age, *GDM* gestational diabetes mellitus, *GWG* gestational weight gain, *LGA* large for gestational age, *MRI* magnetic resonance imaging, *nr* not reported.

*Sub-group analysis on those with overweight/obesity completed.

Of the eight studies published between 2015–2023 [14, 15, 30–35], three were follow-up reports from the Lifestyle study [16, 17, 31–33], two from UPBEAT (UK Pregnancies Better Eating and Activity Trial) [14, 18, 19, 30], one from LiP (Lifestyle in Pregnancy) [22, 35], one from ETIP (Exercise Training in Pregnancy) [20, 21, 34], and one pilot study from ENHANCED (Enhanced Neonatal Health and Neonatal Cardiac Effect Developmentally) [15]. Two RCTs studied ‘the effect of antenatal exercise’ [15, 20], two studied ‘the combined effect of antenatal physical activity and diet’ [18, 22], and one studied ‘the combined effect of preconception physical activity and diet’ [17]. One study also randomised women with normal-weight, but separate analyses enabled examination of the effect of the intervention in women with overweight/obesity [15].

The offspring follow-up period varied between studies: two studies were within the first two months of life [15, 34] and six were at three-to-seven-years-old [14, 30–33, 35]. There were no studies of adolescent or adult offspring.

All studies were sub-samples of the children of women randomised in the original trial and predominantly included around 50–60 offspring but ranged from 18–404. Every study reported difficulties in re-recruiting participants, highlighted by the large attrition rate, with some reporting on <10% of those randomised or eligible.

### Study quality

All studies were ranked as “fair” (susceptible to some bias). There was no evidence of recruitment or randomisation bias in any of the RCTs. The primary limitation of each study was the large attrition rate, which might bias results to those who participated at follow-up. Some studies compared baseline maternal characteristics between those who did and did not participate in the follow-ups, and whilst participants in the follow-up were typically characteristic of the trial population, there were some discrepancies. It is possible that reported differences in age and ethnicity of the women retained at follow-up [14] could impact the success or otherwise of the intervention, but there is no statistical evidence for this provided in the included studies. Only one study undertook sufficient statistical analyses to provide evidence that their results were robust to the presence of missing data: Dalrymple et al. used multivariate imputation chained equations to provide a sample size equivalence to the original UPBEAT population, which provided similar results [30]. The UPBEAT follow-up by Taylor et al. was the only study to provide power calculations for comparison of cardiovascular measures between the trial arms [14], which was likely due to the exploratory nature of the studies. The UPBEAT follow-up by Taylor et al. was also the only study to adjust for offspring body composition in all group-based comparisons, attempting to control for any differences between groups in offspring lifestyle [14]. However, most of the studies included in the review report some outcome measures that have been indexed to body composition, such as left-ventricular mass (LVM) indexed to height.

### Cardiovascular remodelling outcomes

The cardiovascular outcomes that were available for review were cardiac structural and functional measures, heart rate (HR), blood pressure, and measures of arterial thickness and stiffness. The results from individual studies are provided in Tables 3–8.

**Table 3:** Cardiac structure outcomes.

Study (*n* = 5)	Summary (Intervention vs. non-intervention)	Confounders adjusted for in analyses	Results
ENHANCED May, 2023 [15] 4–5 weeks-old *n* = 7 Active (Int: *n* = 3, Non-int: *n* = 4) *n* = 11 Quiet (Int: *n* = 3, Non-int: *n* = 8)	No difference	None considered	Aortic diameter Active Int: 0.97 ± 0.06; Non-int: 0.92 ± 0.06 cm; *p* = 0.93. Quiet Int: 0.97 ± 0.02; Non-int: 0.95 ± 0.18 cm; *p* = 0.23 SV Active Int: 9.49 ± 1.64; Non-int: 9.01 ± 1.20 cm^3^; *p* = 0.48. Quiet Int: 11.72 ± 2.42; Non-int: 9.79 ± 2.52 cm^3^; *p* = 0.76 CO Active Int: 1.47 ± 0.28; Non-int: 1.52 ± 0.22; *p* = 0.77. Quiet Int: 1.58 ± 0.34; Non-int: 1.45 ± 0.35; *p* = 0.78 Cardiac index Active Int: 5.90 ± 1.19; Non-int: 5.74 ± 0.93; *p* = 0.60. Quiet: Int: 6.20 ± 2.03; Non-int: 5.70 ± 1.32; *p* = 0.60
ETIP Nyrnes, 2018 [34] 1–3 days & 6–8 weeks-old *n* = 53 (Int: *n* = 26*, Non-int: *n* = 27) *only *n* = 23 at 6–8 weeks	No difference	None considered	IVSd 1–3 days Int: 5.0 (95% CI 4.5–5.5); Non-int: 5.6 (95% CI 5.1–6.1) mm; 95% CI = −0.6–0.1 6–8 weeks Int: 6.0 (95% CI 5.4–6.5); Non-int: 5.9 (95% CI 5.4–6.4) mm; 95% CI = −0.7–0.9 EDD 1–3 days Int: 1.8 (95% CI 1.8–1.9); Non-int: 1.8 (95% CI 1.7–1.9) mm; 95% CI = −0.1–0.1 *6–8 weeks* Int: 2.2 (95% CI 2.1–2.3); Non-int: 2.2 (95% CI 2.1–2.3) mm; 95% CI = −0.1–0.1
Lifestyle den Harink, 2022 [31] 6.5 years-old *n* = 60 (Int: *n* = 24, Non-int: *n* = 36)	↓Remodelling – reduced IVSd, LVM, LVMi	Group differences: none considered apart from (indexed cardiac measures). Regression analyses: age and sex.	IVSd Int: 5.12 ± 0.70; Non-int: 6.11 ± 0.79 mm; *p* < 0.001 IVSd *z*-score Int: −0.60 ± 0.65; Non-int: 0.27 ± 0.51 mm; *p* < 0.001 LVM Int: 50.0 ± 10.51; Non-int: 58.28 ± 13.40 g; *p* = 0.015 LVMi Int: 53.55 ± 8.52; Non-int: 62.22 ± 8.84 g/m^2^; *p* < 0.001 SV Int: 62.52 ± 14.55; Non-int: 58.13 ± 16.84 mL; *p* = 0.30 CO Int: 5.56 ± 1.24; Non-int: 5.06 ± 1.28 L/min; *p* = 0.14 Other results: The intervention was associated with a reduced IVSd *z*-score (B: −0.88; 95% CI −1.18 to −0.59) and LVMi (B: −8.71; 95% CI −13.20 to −4.22).
Lifestyle den Harink, 2023 [32] 7.1 years-old *n* = 45 (Int: *n* = 18, Non-int: *n* = 27)	↓Remodelling – statistical shape modelling (decreased sphericity and thinner septal wall) No difference – standard metrics of cardiac remodelling	Offspring age and sex. Structural measures indexed to BSA.	EDV Int: 63.03 ± 15.34; Non-int: 64.57 ± 19.91 mL; *p* = 0.78 EDVi Int: 64.80 ± 11.44; Non-int: 65.09 ± 15.47 mL/m^2^; *p* = 0.96 ESV Int: 23.53 ± 7.15; Non-int: 26.83 ± 9.56 mL; *p* = 0.21 ESVi Int: 24.09 ± 5.81; Non-int: 26.99 ± 7.97 mL/m^2^; *p* = 0.19 SV Int: 39.50 ± 9.70; Non-int: 37.74 ± 11.25 mL; *p* = 0.60 IVSd Int: 6.03 ± 0.73; Non-int: 5.96 ± 0.82 mm; *p* = 0.76 IVSdi Int: 6.31 ± 0.95; Non-int: 6.13 ± 1.0 mm/m^2^; *p* = 0.53 LVM Int: 40.02 ± 6.74; Non-int: 41.13 ± 10.41 g; *p* = 0.68 LVMi Int: 41.40 ± 4.85; Non-int: 41.56 ± 7.68 g/m^2^; *p* = 0.96 Statistical shape modelling captured a significant (*p* = 0.023) pointier LV shape (i.e. decreased sphericity) and a thinner septal wall, most prominent in the posterior-septal region, in the intervention group.
UPBEAT Taylor, 2022 [14] 3.7 years-old *n* = 69 (Int: *n* = 30, Non-int: *n* = 39)	↓Remodelling – decreased IVSd, PWd, RWT, LVM/EDV ↑Remodelling – increased SV	Maternal ethnicity and smoking status at baseline & offspring age, sex, and BMI z-score.	EDV Int: 33.2 ± 6.5; Non-int: 30.8 ± 4.8 mL; *p* = 0.81 (unadjusted), *p* = 0.22 (adjusted) ESV Int: 11.4 ± 2.6; Non-int: 11.3 ± 2.2 mL; *p* = 0.91 (unadjusted), *p* = 0.77 (adjusted) SV Int: 21.8 ± 4.8; Non-int: 19.5 ± 3.4 mL; *p* = 0.021 (unadjusted), *p* = 0.072 (adjusted) CO Int: 2.20 ± 0.45; Non-int: 2.03 ± 0.41 L/min; *p* = 0.11 (unadjusted), *p* = 0.32 (adjusted) EDD Int: 3.20 ± 0.23; Non-int: 3.14 ± 0.24 cm; *p* = 0.32 (unadjusted), *p* = 0.61 (adjusted) ESD Int: 2.02 ± 0.21; Non-int: 2.02 ± 0.21 cm; *p* = 0.90 (unadjusted), *p* = 0.46 (adjusted) IVSd Int: 0.45 ± 0.05; Non-int: 0.47 ± 0.04 cm; *p* = 0.026 (unadjusted), *p* = 0.008 (adjusted) PWd Int: 0.45 ± 0.04; Non-int: 0.48 ± 0.05 cm; *p* = 0.019 (unadjusted), *p* = 0.005 (adjusted) RWT Int: 0.28 ± 0.03; Non-int: 0.30 ± 0.04; *p* = 0.013 (unadjusted), *p* = 0.012 (adjusted) LVM Int: 31.0 ± 5.6; Non-int: 32.0 ± 4.9 g; *p* = 0.43 (unadjusted), *p* = 0.13 (adjusted) LVMi Int: 30.7 ± 4.5; Non-int: 32.4 ± 5.5 g/m^2.7^; *p* = 0.16 (unadjusted), *p* = 0.065 (adjusted) LVM/EDV Int: 0.95 ± 0.18; Non-int: 1.06 ± 0.20 g/mL; *p* = 0.024 (unadjusted), *p* = 0.028 (adjusted) LAV Int: 14.8 ± 4.3; Non-int: 15.6 ± 4.4 mL; *p* = 0.43 (unadjusted), *p* = 0.36 (adjusted) LAVi Int: 21.7 ± 6.1; Non-int: 23.1 ± 6.1 mL/m^2^; *p* = 0.34 (unadjusted), *p* = 0.34 (adjusted)

*BMI* body mass index, *BSA* body surface area, *CO* cardiac output, *EDD* left-ventricular end-diastolic diameter, *EDV* left-ventricular end-diastolic volume, *ESV* left-ventricular end-systolic diameter, *Int* intervention group, *IVSd* intraventricular septal thickness at end-diastole, *IVSdi* IVSd indexed to BSA, *LAV* left-atrial volume, *LAVi* left-atrial volume indexed to a power of height, *LVM* left-ventricular mass, *LVMi* left-ventricular mass indexed to a power of height or BSA, *Non-int* non-intervention group, *PWd* posterior-wall thickness at end-diastole, *RWT* relative wall thickness, *SV* stroke volume.

**Table 4:** Cardiac systolic function outcomes.

Study (*n* = 5)	Summary (Intervention vs. non-intervention)	Confounders adjusted for in analyses	Results
ENHANCED May, 2023 [15] 4–5 weeks-old *n* = 7 Active (Int: *n* = 3, Non-int: *n* = 4) *n* = 11 Quiet (Int: *n* = 3, Non-int: *n* = 8)	↑ Function – increased FS in active offspring	None considered	LV EF Active Int: 74.5 ± 1.4; Non-int: 66.5 ± 5.1%; *p* = 0.23. Quiet Int: 69.8 ± 3.6; Non-int: 67.6 ± 4.3%; *p* = 0.36 LV FS Active Int: 39.5 ± 2.9; Non-int: 38.5 ± 8.1%; *p* = 0.03. Quiet Int: 35.3 ± 2.5; Non-int: 36.7 ± 5.0%; *p* = 0.55
ETIP Nyrnes, 2018 [34] 1–3 days & 6–8 weeks-old *n* = 53 (Int: *n* = 26*, Non-int: *n* = 27) *only *n* = 23 at 6–8 weeks	No difference	None considered	LV FS 1–3 days Int: 37.0 (95% CI 35.0–39.0); Non-int: 36.2 (95% CI 34.2–38.1) %; 95% CI = −2.0–3.6 6–8 weeks Int: 35.2 (95% CI 33.1–37.3); Non-int: 35.0 (95% CI 33.0–37.0) %; 95% CI = −2.8–3.1 LV GLS 1–3 days Int: −17.2 (95% CI −18.5–15.9); Non-int: −16.9 (95% CI −18.1–15.6) %; 95% CI = −2.1–1.5 6–8 weeks Int: −21.0 (95% CI −22.4–19.7); Non-int: −20.1 (95% CI −21.4–18.9) %; 95% CI = −2.7–0.9 LV GLSR 1–3 days Int: −1.6 (95% CI −1.7–1.6); Non-int: −1.7 (95% CI −1.7–1.6); 95% CI = −0.1–0.2 6–8 weeks Int: −1.8 (95% CI −1.9–1.7); Non-int: −1.8 (95% CI −1.9–1.7); 95% CI = −0.2–0.2 MAPSE 1–3 days Int: 3.9 (95% CI 3.6–4.2); Non-int: 3.9 (95% CI 3.6–4.2) mm; 95% CI = −0.4–0.4 6–8 weeks Int: 6.5 (95% CI 6.2–6.8); Non-int: 6.3 (95% CI 6.0–6.6) mm; 95% CI = −0.2–0.6 S’ (average of septal & lateral walls) 1–3 days Int: 4.4 (95% CI 3.9–4.8); Non-int: 4.2 (95% CI 3.7–4.6) cm/s; 95% CI = −0.4–0.8 6–8 weeks Int: 6.1 (95% CI 5.6–6.6); Non-int: 6.2 (95% CI 5.7–6.6) cm/s; 95% CI = −0.7–0.6 RV GLS 1–3 days Int: −20.9 (95% CI −23.0–18.9); Non-int: −18.6 (95% CI −20.6–16.7) %; 95% CI = −5.1–0.6 6–8 weeks Int: −22.7 (95% CI −24.9–20.5); Non-int: −21.3 (95% CI −23.3–19.3) %; 95% CI = −4.4–1.5 RV GLSR 1–3 days Int: −1.9 (95% CI −2.1–1.7); Non-int: −1.7 (95% CI −2.0–1.5); 95% CI = −0.5–0.2 6–8 weeks Int: −2.5 (95% CI −2.8–2.2); Non-int: −2.1 (95% CI −2.4–1.9); 95% CI = −0.7–0.0 TAPSE 1–3 days Int: 9.3 (95% CI 8.6–9.9); Non-int: 9.0 (95% CI 8.3–9.6) mm; 95% CI = −0.6–1.2 6–8 weeks Int: 13.9 (95% CI 13.2–14.6); Non-int: 14.2 (95% CI 13.6–14.9) mm; 95% CI = −1.3–0.6
Lifestyle den Harink, 2022 [31] 6.5 years-old *n* = 60 (Int: *n* = 24, Non-int: *n* = 36)	↑ Function – increased lateral wall S’	Lateral wall S’ adjusted for offspring age, sex, and BSA in regression analyses. Not considered for other measures.	LV EF Int: 54.44 ± 4.78; Non-int: 55.43 ± 3.52%; *p* = 0.34 LV GLS Int: −23.82 ± 3.44; Non-int: −24.25 ± 2.55%; *p* = 0.61 Septal S’ Int: 6.79 ± 1.09; Non-int: 6.41 ± 0.99 cm/s; *p* = 0.17 Lateral S’ Int: 7.27 ± 1.74; Non-int: 5.87 ± 1.3 cm/s; *p* = 0.001 RV S’ Int: 10.39 ± 1.91; Non-int: 10.02 ± 2.26 cm/s; *p* = 0.52 Other results: When adjusted for age, sex, and BSA, the intervention was associated with a 1.5 (0.7–2.2) cm/s higher lateral wall S’.
Lifestyle den Harink, 2023 [32] 7.1 years-old *n* = 45 (Int: *n* = 18, Non-int: *n* = 27)	↑ Function – increased EF	Offspring age and sex	LV EF Int: 63.02 ± 6.18; Non-int: 58.78 ± 5.77%; *p* = 0.02
UPBEAT Taylor, 2022 [14] 3.7 years-old *n* = 69 (Int: *n* = 30, Non-int: *n* = 39)	↑ Function – increased EF. Some evidence of increased GLS and lateral wall S’	Maternal ethnicity and smoking status at baseline & offspring age, sex, and BMI *z*-score.	LV EF Int: 65.6 ± 4.9; Non-int: 63.1 ± 4.9%; *p* = 0.042 (unadjusted), *p* = 0.063 (adjusted) LV FS Int: 36.7 ± 4.6; Non-int: 35.3 ± 5.8%; *p* = 0.26 (unadjusted), *p* = 0.17 (adjusted) LV GLS Int: −18.1 ± 1.9; Non-int: −17.6 ± 2.0%; *p* = 0.30 (unadjusted), *p* = 0.085 (adjusted) Lateral S’ Int: 0.09 ± 0.01; Non-int: 0.08 ± 0.01 m/s; *p* = 0.066 (unadjusted), *p* = 0.10 (adjusted)

*BMI* body mass index, *BSA* body surface area, *EF* ejection fraction, *FS* fractional shortening, *GLS* global longitudinal strain, *GLSR* global longitudinal strain rate, *Int* intervention group, *LV* left-ventricular, *MAPSE* mitral valve annular plane systolic excursion, *Non-int* non-intervention group, *RV* right-ventricular, *S’* longitudinal peak systolic myocardial velocity, *TAPSE* tricuspid valve annular plane systolic excursion.

**Table 5:** Cardiac diastolic function outcomes.

Study (*n* = 3)	Summary (Intervention vs. non-intervention)	Confounders adjusted for in analyses	Results
ETIP Nyrnes, 2018 [34] 1–3 days & 6–8 weeks-old *n* = 53 (Int: *n* = 26*, Non-int: *n* = 27) *only *n* = 23 at 6–8 weeks	No difference	None considered	Diastolic function e’ (average of septal & lateral walls) 1–3 days Int: 5.8 (95% CI 5.1–6.5); Non-int: 5.5 (95% CI 4.7–6.2) cm/s; 95% CI = −0.7–1.4 6–8 weeks Int: 9.6 (95% CI 8.8–10.4); Non-int: 8.8 (95% CI 8.1–9.6) cm/s; 95% CI = −0.3–1.8 a’ (average of septal & lateral walls) 1–3 days Int: 6.1 (95% CI 5.4–6.8); Non-int: 6.4 (95% CI 5.7–7.0) cm/s; 95% CI = −1.2–0.7 6–8 weeks Int: 8.6 (95% CI 7.9–9.4); Non-int: 8.5 (95% CI 7.8–9.2) cm/s; 95% CI = −0.9–1.1
Lifestyle den Harink, 2022 [31] 6.5 years-old *n* = 60 (Int: *n* = 24, Non-int: *n* = 36)	↑ Function – increased lateral wall e’. Some evidence of increased septal wall e’ and reduced septal wall E/e’.	Lateral wall e’ adjusted for offspring age, sex, and BSA in regression analyses. Not considered for other measures.	Diastolic function E/A Int: 2.03 (1.41–4.97); Non-int: 2.29 (1.34–4.56) cm/s; *p* = 0.63 E/e’ Int: 5 ± 1; Non-int: 6 ± 2; *p* = 0.09 Septal e’ Int: 13.82 ± 1.99; Non-int: 12.87 ± 1.99 cm/s; *p* = 0.08 Septal a’ Int: 5.68 ± 1.21; Non-int: 5.59 ± 1.76 cm/s; *p* = 0.84 Lateral e’ Int: 17.78 ± 2.99; Non-int: 15.59 ± 3.34 cm/s; *p* = 0.012 Lateral a’ Int: 6.91 (3.72–10.29); Non-int: 5.92 (3.05–16.92) cm/s; *p* = 0.09 Lateral e’/a’ Int: 2.83 ± 0.85; Non-int: 2.90 ± 1.14; *p* = 0.80 RV e’ Int: 14.26 ± 3.34; Non-int: 13.57 ± 2.55 cm/s; *p* = 0.37 RV a’ Int: 9.74 ± 2.81; Non-int: 8.65 ± 1.92 cm/s; *p* = 0.08 Other results: When adjusted for age, sex, and BSA, the intervention was associated with a 2.3 (0.6–4.0) cm/s higher lateral wall e’.
UPBEAT Taylor, 2022 [14] 3.7 years-old *n* = 69 (Int: *n* = 30, Non-int: *n* = 39)	Some evidence of increased E/A.	Maternal ethnicity and smoking status at baseline & offspring age, sex, and BMI z-score.	Diastolic function E/A Int: 1.78 ± 0.49; Non-int: 1.58 ± 0.35; *p* = 0.067 (adjusted), *p* = 0.10 (adjusted) Lateral e’ Int: 0.15 ± 0.02; Non-int: 0.14 ± 0.02 m/s; *p* = 0.35 (adjusted), *p* = 0.44 (adjusted) Lateral a’ Int: 0.068 ± 0.01; Non-int: 0.074 ± 0.02 m/s; *p* = 0.24 (adjusted), *p* = 0.38 (adjusted) Lateral E/e’ Int: 6.9 ± 1.8; Non-int: 6.8 ± 1.3; *p* = 0.85 (adjusted), *p* = 0.71 (adjusted)

*a’* peak longitudinal late myocardial tissue velocities, *BMI* body mass index, *BSA* body surface area, *e’* peak longitudinal early myocardial tissue velocities, *E/A* early-to-late peak mitral inflow velocities ratio, *e’/a’* e’-to-a’ ratio, *Int* intervention group, *Non-int* non-intervention group.

**Table 6:** Heart rate outcomes.

Study (*n* = 6)	Summary (Intervention vs. non-intervention)	Confounders adjusted for in analyses	Results
ENHANCED May, 2023 [15] 4–5 weeks-old *n* = 7 Active (Int: *n* = 3, Non-int: *n* = 4) *n* = 11 Quiet (Int: *n* = 3, Non-int: *n* = 8)	Some evidence of decreased heart rate	None considered	*Active* Int: 155.7 ± 28.7; Non-int: 168.5 ± 12.8 bpm; *p* = 0.13 Quiet Int: 134.3 ± 5.1; Non-int: 149.4 ± 12.1 bpm; *p* = 0.12 Other results: Infant activity state (B = −0.44, *p* = 0.006) and maternal pregnancy exercise level (B = 0.49, *p* = 0.01) were associated with offspring resting heart rate (r^2^ = 0.40, *p* = 0.003).
ETIP Nyrnes, 2018 [34] 1–3 days & 6–8 weeks-old *n* = 53 (Int: *n* = 26*, Non-int: *n* = 27) *only *n* = 23 at 6–8 weeks	No statistics (possibly decreased at 6-8 weeks)	None considered	1–3 days Int: 123 (95% CI: 116–129); Non-int: 122 (95% CI: 115–128) bpm; statistics not reported 6–8 weeks Int: 143 (95% CI: 136–151); Non-int: 148 (95% CI: 141–154) bpm; statistics not reported
Lifestyle den Harink, 2022 [31] 6.5 years-old *n* = 60 (Int: *n* = 24, Non-int: *n* = 36)	No difference	Not considered for heart rate	Int: 89.3 ± 7.3; Non-int: 88.6 ± 12.1 bpm; *p* = 0.80
Lifestyle den Harink, 2023 [32] 7.1 years-old *n* = 45 (Int: *n* = 18, Non-int: *n* = 27)	No difference	Offspring age and sex	Int: 87.1 ± 9.6; Non-int: 90.8 ± 18.1 bpm; *p* = 0.27
UPBEAT Dalrymple, 2021 [30] 3.5 years-old *n* = 403 (Int: *n* = 199, Non-int: *n* = 204)	↓ Decreased heart rate	Maternal BMI, parity and ethnicity & offspring age and sex	Int: 91 ± 20; Non-int: 96 ± 17 bpm; *p* = 0.01 Other results: Sensitivity analyses using multiple imputation for the whole trial population demonstrated a consistent reduction of resting pulse rate in the intervention arm (−4.8 bpm [95% CI −8.37 to −1.23]).
UPBEAT Taylor, 2022 [14] 3.7 years-old *n* = 70 (Int: *n* = 31, Non-int: *n* = 39)	No difference	Maternal ethnicity and smoking status at baseline & offspring age, sex, and BMI z-score	Minimum heart rate Int: 87 ± 10.2; Non-int: 90 ± 10.8 bpm; *p* = 0.71 (unadjusted), *p* = 0.72 (adjusted) Maximum heart rate Int: 129 ± 9.0; Non-int: 132 ± 14.0 bpm; *p* = 0.47 (unadjusted), *p* = 0.51 (adjusted) SDNN – HRV Int: 32.8 ± 10.5; Non-int: 32.8 ± 10.8 ms; *p* = 0.98 (unadjusted), *p* = 0.70 (adjusted) RMSSD – HRV Int: 32.0 ± 13.5; Non-int: 32.7 ± 15.1 ms; *p* = 0.84 (unadjusted), *p* = 0.88 (adjusted) pNN50 – HRV Int: 13.7 ± 22.8; Non-int: 14.3 ± 12.3%; *p* = 0.70 (unadjusted), *p* = 0.98 (adjusted)

*BMI* body mass index, *bpm* beats per minute, *Int* intervention group, *Non-int* non-intervention group, *pNN50* proportion of the number of pairs of successive NN (R-R) intervals that differ by more than 50 ms, *RMSSD* root mean square of the successive differences, *SDNN* standard deviation of the NN (R-R) intervals.

**Table 7:** Blood pressure outcomes.

Study (*n* = 5)	Summary (Intervention vs. non-intervention)	Confounders adjusted for in analyses	Results
ETIP Nyrnes, 2018 [34] 1–3 days & 6–8 weeks-old *n* = 53 (Int: *n* = 26*, Non-int: *n* = 27) *n = 23 at 6–8 weeks	No difference	None considered	sBP (1–3 days) Int: 80 (95% CI: 74–85); Non-int: 82 (95% CI: 77–87) mmHg; *p* = not reported dBP (1–3 days) Int: 46 (95% CI: 41–50); Non-int: 47 (95% CI: 43–51) mmHg; *p* = not reported sBP (6–8 weeks) Int: 83 (95% CI: 78–89); Non-int: 88 (95% CI: 83–93) mmHg; *p* = not reported dBP (6–8 weeks) Int: 51 (95% CI: 46–55); Non-int: 52 (95% CI: 40–56) mmHg; *p* = not reported
Lifestyle Mintjens, 2021 [33] 4.6 years-old *n* = 43 (Int: *n* = 16, Non-int: *n* = 27)	No difference	Offspring sex (supplement analyses)	sBP *z*-score Int: 0.46 ± 0.65 (*n* = 16); Non-int: 0.54 ± 0.57 (*n* = 27); 95% CI = −0.46–0.30 dBP *z*-score Int: 0.91 ± 0.66 (*n* = 16); Non-int: 0.96 ± 0.57 (*n* = 27); 95% CI = −0.44–0.33
LiP Tanvig, 2015 [35] 2.8 years-old *n* = 150 (Int: *n* = 77, Non-int: *n* = 73)	No difference	None considered for blood pressure	sBP Int: 98.3 (93.7–105.3); Non-int: 97.3 (94.3–101.3) mmHg; *p* = not reported dBP Int: 64.3 (61.0–67.3); Non-int: 62.0 (60.3–65.3) mmHg; *p* = not reported
UPBEAT Dalrymple, 2021 [30] 3.5 years-old *n* = 404 (Int: *n* = 197, Non-int: *n* = 207) *Int: *n* = 196 & Non-int: *n* = 205 for dBP	No difference	Maternal BMI, parity and ethnicity & offspring age and sex	sBP percentile Int: 80 (63–91); Non-int: 78 (63–90) %; *p* = 0.23 dBP percentile Int: 79 (57–91); Non-int: 82 (64–88) mmHg; *p* = 0.22
UPBEAT Taylor, 2022 [14] 3.7 years-old *n* = 70 (Int: *n* = 31, Non-int: *n* = 39)	No difference	Maternal ethnicity and smoking status at baseline & offspring age, sex, and BMI z-score	sBP Int: 83 (68–88); Non-int: 78 (61–82) mmHg *p* = 0.36 (unadjusted), *p* = 0.29 (adjusted) dBP Int: 75 (65–85); Non-int: 70 (53–83) % *p* = 0.52 (unadjusted), *p* = 0.89 (adjusted)

*BMI* body mass index, *dBP* diastolic blood pressure, *Int* intervention group, *Non-int* non-intervention group, *sBP* systolic blood pressure.

**Table 8:** Arterial stiffening outcomes.

Study (*n* = 4)	Summary (Intervention vs. non-intervention)	Confounders adjusted for in analyses	Results
Lifestyle Mintjens, 2021 [33] 4.6 years-old *n* = 34 (Int: *n* = 12, Non-int: *n* = 22)	No difference	Offspring sex (supplement analyses)	PWV Int: 4.51 ± 0.83; Non-int: 4.50 ± 1.14 m/s; 95% CI = −0.75–0.70
Lifestyle den Harink, 2022 [31] 6.5 years-old *n* = 60 (Int: *n* = 17, Non-int: *n* = 26)	No difference	None considered for CIMT	Left CIMT Int: 0.49 ± 0.04; Non-int: 0.47 ± 0.07 mm; *p* = 0.36 Right CIMT Int: 0.46 ± 0.04; Non-int: 0.47 ± 0.05 mm; *p* = 0.29
Lifestyle den Harink, 2023 [32] 7.1 years-old *n* = 41 (Int: *n* = 15, Non-int: *n* = 26)	No difference	Offspring age and sex	PWV Int: 2.66 ± 0.87; Non-int: 2.41 ± 0.89 cm/ms; *p* = 0.36
UPBEAT Taylor, 2022 [14] 3.7 years-old *n* = 69 (Int: *n* = 30, Non-int: *n* = 39)	No difference	Maternal ethnicity and smoking status at baseline & offspring age, sex, and BMI z-score	PWV Int: 4.6 ± 2.1; Non-int: 4.3 ± 1.5 m/s; *p* = 0.53 (unadjusted), *p* = 0.53 (adjusted) CIMT Int: 0.47 ± 0.04; Non-int: 0.47 ± 0.04 mm; *p* = 0.99 (unadjusted), *p* = 0.64 (adjusted)

*BMI* body mass index, *CIMT* carotid intima media thickness, *Int* intervention group, *Non-int* non-intervention group, *PWV* pulse-wave velocity.

### Cardiac structural remodelling

Five studies reported data on cardiac structure in 245 offspring [14, 15, 31, 32, 34]. RCTs appeared to limit cardiac structural remodelling in the intervention versus non-intervention group (Table 3).

The UPBEAT RCT was an antenatal diet and physical activity intervention in 1555 women with the aim of reducing glycaemic load [18, 19]. A small sub-sample of the UPBEAT trial (*n* = 70; <10% of the original population) was followed-up at three years-old [14]. In support of the previous systematic reviews [4, 10, 11], Taylor et al. reported that the standard care (non-intervention) UPBEAT offspring had evidence of cardiac structural remodelling compared to offspring of non-randomised normal-weight mothers [14]. Those in the intervention when compared to those in the non-intervention arm had reduced interventricular-septum (MD = −0.02 [−0.04, −0.002] cm; *p* = 0.026) and posterior wall (MD = −0.02 [−0.05, −0.005] cm; *p* = 0.019) diameters at end-diastole (IVSd and PWd, respectively), reduced relative wall thickness (RWT: MD = −0.02 [−0.04, −0.005]; *p* = 0.013), reduced LVM to end-diastolic volume (EDV) ratio (LVM/EDV: MD = −0.11 [−0.20, −0.01]; *p* = 0.024), and a higher stroke volume (SV: MD = 2.3 [0.35, 4.33] mL; *p* = 0.021) all assessed by echocardiography. These differences remained, and for some outcome measures, were strengthened after adjustment for relevant confounders. A lower LVM indexed to height^2.7^, after adjustment for confounders, did not reach statistical significance (MD = −2.45 [−5.06, 0.16] g/m^2.7^; *p* = 0.065) [14].

The Lifestyle study was the only intervention RCT to commence in the preconception period and was a study of sub-fertile women with obesity. The intervention consisted of physical activity and a dietary intervention designed to reduce body weight by 5–10% before conception [16, 17]. Similar to UPBEAT, den Harink et al. used echocardiography to measure cardiac remodelling in 6–7-year-old offspring of the Lifestyle trial and identified reduced remodelling in IVSd (MD = −0.99 [−1.58, −0.40] mm; *p* < 0.001), IVSd *z*-score (MD = −0.87 [−1.17, −0.57]; *p* < 0.001), LVM (MD = −8.28 [−14.95, −1.61] g; *p* = 0.015), and LVM indexed to height^2^ (MD = −8.67 [−13.30, −4.05] g/m^2^; *p* < 0.001) [31]. These results persisted after adjustment for child body surface area (BSA), age, and sex. A sub-sample of the echocardiography cohort also had cardiac magnetic resonance imaging (MRI) performed, but there were no differences in standard metrics of cardiac remodelling between groups using this method [32]. However, den Harink et al. also undertook statistical shape modelling (SSM) of the cardiac MRI images, which provides novel insights into cardiac remodelling whereby standard metrics cannot. In SSM, a common geometrical template is used to describe left-ventricular anatomy of each subject and principal component analysis is then undertaken to identify the key modes of variation [36–38]. The authors identified a 3D pattern of reduced IVSd thickening, similar to that observed in the UPBEAT and Lifestyle echocardiography studies, and reduced left-ventricular sphericity in the intervention arm [32]. Limitations were that 10.5% of the women in the non-intervention group lost >5% body weight in the first six-months, potentially suggesting some ‘treatment’ contamination, and only 43% of the intervention group who completed the intervention (intervention and infertility treatment) achieved the target weight loss of 5–10%. Also, the follow-up sample consisted of <20% of the original population [16].

May et al. randomised 140 women of any weight classification in a pilot study to either moderate-intensity exercise or low-intensity stretching at 13–16 weeks gestational age [15]. Fifty-six neonates were available for follow-up, but only 18 were born to women with overweight or obesity. Amongst these, there was no difference in cardiac structure associated with the maternal intervention. This study was limited by a very small sample-size of those born to mothers with overweight/obesity, with some arms having only three participants [15].

Nyrnes et al. followed-up 53 offspring of the ETIP trial which consisted of four-times weekly antenatal exercise [20, 21, 34]. There was no difference in cardiac structure associated with the maternal intervention. Limitations were that maternal adherence to the intervention was only 50% [21].

### Cardiac functional remodelling

Five studies reported data on cardiac function [14, 15, 31, 32, 34]. All five reported systolic function (*n* = 245), but only three reported diastolic function (*n* = 182) [14, 31, 34].

### Systolic cardiac function

RCTs appeared to improve some measures of systolic function in the intervention group compared to the non-intervention group (Table 4).

The two follow-up studies of the Lifestyle study identified better systolic function. The echocardiography study identified better lateral wall peak longitudinal systolic velocity (S’: MD = 1.42 [0.63, 2.20] cm/s; *p* = 0.001), which remained when adjusted for BSA, age, and sex. There were no differences in septal wall or right-ventricular S’, left-ventricular ejection fraction (EF), or left-ventricular global longitudinal strain (GLS) [31]. The cardiac MRI follow-up identified improved left-ventricular EF (MD = 4.24 [0.67, 7.81] %; *p* = 0.02) [32].

In the follow-up of UPBEAT offspring by Taylor et al., there was an increase in left-ventricular EF (MD = 2.5 [0.09, 4.91] %; *p* = 0.042). Increased lateral wall S’ (MD = 0.01 [−0.0007, 0.02] m/s; *p* = 0.066) did not reach statistical significance. There were no differences in GLS or fractional-shortening (FS) [14].

May et al. identified better left-ventricular FS in ‘active’ (MD = 1.0 [0.10, 1.90] %; *p* = 0.03), but not ‘quiet’ neonates of the intervention arm, which could be an artifact of neonate movement and a small sample size or could indicate better systolic function during physiological stress. There were no differences in left-ventricular EF. This study was severely limited by the small sample sizes described above [15].

Nyrnes et al. did not find any differences in systolic function between intervention arms, but a limitation of this study was poor adherence to the intervention [21, 34].

### Diastolic cardiac function

Although limited to three studies and only 182 participants in total, there was some evidence of better diastolic function in offspring exposed to maternal interventions compared to the non-intervention arm (Table 5).

Using echocardiography, den Harink et al. reported that offspring in the intervention arm had increased left-ventricular lateral wall peak longitudinal early myocardial tissue velocity (e’: MD = 2.26 [0.56, 3.96] cm/s; *p* = 0.012), which remained when adjusted for age, sex, and BSA. A higher septal wall e’ (MD = 0.95 [−0.11, 2.01] cm/s; *p* = 0.08) and a lower peak early mitral inflow velocity (E) to e’ ratio (E/e’: MD = −1.0 [−2.16, 0.16]; *p* = 0.09) did not reach statistical significance [31].

The increased early-to-late mitral filling patterns in the UPBEAT follow-up by Taylor et al. did not reach statistical significance (E/A ratio: MD = 0.18 [−0.01, 0.39]; *p* = 0.067). There were no differences in lateral wall tissue Doppler imaging metrics [14].

Although the follow-up of the ETIP cohort found increased e’ velocities at 6–8 weeks, this did not reach statistical significance (MD = 0.8 [−0.3, 1.8] cm/s; *p*-value not reported) [34].

### Heart rate

Six studies reported data on HR in 649 offspring [14, 15, 30–32, 34]. There was some evidence to suggest a decreased HR in the intervention versus non-intervention group (Table 6).

Dalrymple et al. investigated a sub-sample of children from the UPBEAT cohort (*n* = 403) and reported a 5 (−8.41, −1.07; *p* = 0.01) beats-per-minute (bpm) lower HR in children (3 years) born to mothers in the intervention versus the non-intervention arm [30]. This was the follow-up study with the largest sample size and the only one to account for missing data; when sensitivity analyses using multiple imputations were performed, the five bpm reduction in HR with intervention persisted, providing reassurance that the results were not due to selection bias or missing data [30]. In a sub-sample of the study by Dalrymple et al. [30], Taylor et al. [14] identified that whilst children in the UPBEAT non-intervention arm had significantly increased minimum, maximum and mean HRs compared to children of normal BMI mothers, there was no difference between the intervention and non-intervention arms. Taylor et al. also examined the effect of HR variability, but the intervention had no effect on any of the reported outcomes [14].

Although the >12 bpm lower HR in the intervention versus non-intervention group did not reach statistical significance in the follow-up study by May et al., there was a relationship between maternal pregnancy exercise level and offspring resting heart rate (B = 0.49 bpm/metabolic equivalent minutes per week; *p* = 0.01). This study was limited by the very small sample size of mothers with overweight/obesity [15].

Nyrnes et al. identified a five-bpm reduction in HR at 6–8 weeks-old in the intervention group, but no inter-group statistical analyses were performed [34]. This study was limited by the low adherence detailed above.

Both follow-ups of the Lifestyle study by den Harink et al. did not find any differences HR between the two groups [31, 32]. These studies limitations are described above.

### Blood pressure

Five studies reported data on blood pressure in 720 offspring [14, 30, 33–35]. There were no differences in systolic or diastolic blood pressure between the intervention or non-intervention groups in any of the studies (Table 7).

### Arterial stiffness

Four studies reported data on arterial thickness and/or pulse-wave velocity in 204 offspring (a proxy for arterial stiffness) [14, 31–33]. There were no differences in either arterial wall thickness or measures of pulse-wave velocity between the intervention or non-intervention groups in any study (Table 8).

## Discussion

**Fig. 2:**
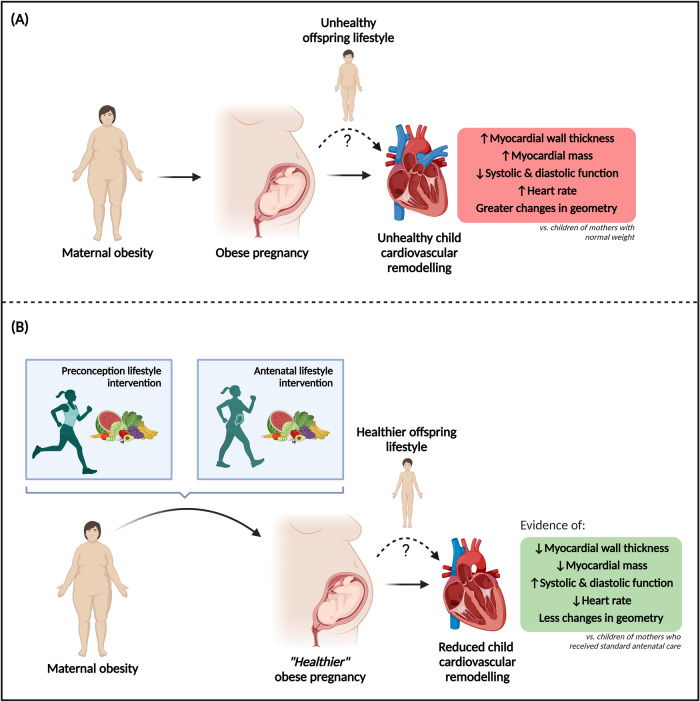
Child offspring cardiovascular health benefits of preconception and antenatal lifestyle interventions in women with obesity. The impact of maternal obesity on child cardiovascular remodelling (**A**) and the protective effect of preconception and antenatal lifestyle interventions (**B**). Figure created with BioRender.com.

We have systematically reviewed whether preconception or antenatal lifestyle interventions in mothers with obesity can lead to a healthier cardiovascular remodelling pattern in their offspring. Eight follow-up studies from five RCTs were identified, providing data on offspring until seven-years-of-age. Although all studies experienced large attrition rates and relatively small sample sizes, potentially limiting statistical power, we identified some evidence of a protective effect of maternal preconception or antenatal lifestyle interventions on offspring cardiovascular remodelling (Fig. 2). While these sub-clinical findings are limited to childhood, this reduction in cardiovascular remodelling, if persisting until adulthood, could incur protection against the adverse cardiovascular outcomes experienced by adult offspring of women with obesity [8, 9].

### Summary of findings

Maternal obesity is associated with offspring cardiac structural remodelling, with increased interventricular-septal thickening commonly reported [11, 12, 14]. In this review, we found that maternal lifestyle interventions appeared to limit this remodelling with reduced interventricular-septal thickness consistently identified in the intervention arms. Increased septal thickening has been identified in conditions associated with increased cardiovascular disease risk, such as childhood obesity [37] and early hypertension [39, 40]. Although it is unclear whether childhood cardiovascular remodelling has pathological implications, increased IVSd may be a predictor of future cardiovascular disease risk [41].

Reduced interventricular-septal thickness was also reported when SSM methods were used. As described in the results, SSM provides novel insights into cardiac remodelling where standard metrics cannot [36–38]. Indeed, the findings of den Harink et al. suggest that reduced interventricular-septal thickness can be identified in the intervention group, even in the absence of any differences in standard metrics [32], highlighting the potential of this method for identifying early changes in cardiac structure.

An increase in left-ventricular sphericity by SSM has been reported in children with obesity [37], which might be a physiological response to normalise increased left-ventricular wall stress [42]. den Harink et al. identified reduced left-ventricular sphericity in the intervention arm [32]. In the UPBEAT neonates, more spherical left-ventricles were identified in neonates of mothers with normal-weight versus mothers with obesity [13], which paradoxically seems to contrast with den Harink et al. Interpretation is therefore difficult as to *how* and *why* the sphericity of the left-ventricle changes in response to maternal obesity and preconception/antenatal lifestyle interventions. For example, babies born preterm have been shown to display more “globular” left ventricles compared to a more “conical” geometry in babies born at term, and this difference is significantly reduced at three-months of age [43]. The conflicting data could thus theoretically be explained by the age difference between studies (newborns versus 6–7-years-olds). Another confounding factor lies in the different definitions and interpretations of shape sphericity, especially when derived from SSM built from small datasets (*n* = 33 and *n* = 45) [13, 32]. It is recognised however that an increment in left-ventricular sphericity is associated with adverse cardiovascular outcomes in adults [44]. Further investigation of the impact of maternal obesity and preconception/antenatal lifestyle interventions on left-ventricular geometric remodelling as assessed by SSM are warranted.

In addition to structural evaluation, functional cardiac measures provide insights into cardiac remodelling patterns. Previous studies indicate that maternal obesity may cause reduced systolic and diastolic cardiac function in the offspring [11, 14, 45]. We report some evidence that maternal lifestyle interventions may protect against any early systolic or diastolic functional impairments, independent of BMI or BSA [14, 31], but investigations with larger sample sizes are needed to corroborate these results. Furthermore, whilst it is established that cardiac remodelling in early adulthood serves as a predictor for future cardiovascular events [46, 47], it remains to be determined whether these early functional impairments observed in offspring of mothers with obesity may play a role in the adverse outcomes reported in adults [8, 9].

The study by den Harink et al. identified increased e’ velocities in the intervention arm, but also found some evidence of increased a’ velocities, that may indicate impaired diastolic function by an increased reliance on atrial filling to expand the ventricle. However, an increase in both suggests no overall change in the pattern of myocardial relaxation, corroborated by the similar ratio of e’ to a’ (e’/a’) reported by den Harink et al. [31]. The e’/a’ ratio is strongly related with obesity in the young [48]. As the e’/a’ ratio was not consistently reported by the other included studies, it is difficult to interpret the relative contribution of early or late ‘relaxation’ for ventricular filling. Other measures of diastolic function, such as diastolic strain rate, as well as other measures of systolic function, such as first-phase EF [49], should also be utilised in future follow-ups.

An increased HR is predictive of future cardiovascular events [50]. There is some evidence to suggest that children born to mothers with obesity have higher resting HRs [12, 14], which might predispose them to elevated risk when exposed to additional environmental stress. Some studies reported reduced HR in offspring following maternal lifestyle interventions, but replication is required, especially given the lack of difference between intervention arms in animal studies [6].

We identified no evidence for a reduction in blood pressure or arterial stiffness in offspring from the RCTs. Exercise in pregnant mice with obesity has also shown no effect on offspring blood pressure, despite an effect on cardiac structure and function [6]. Many years of sustained exposure to an unhealthy lifestyle in the offspring may be needed before a demonstrable impact on vascular stiffness, and subsequent elevation of blood pressure [51, 52].

### Mechanisms

It is likely that multiple biological pathways underpin relationships between maternal obesity and childhood cardiovascular function and the potential benefit of preconception and antenatal lifestyle interventions [53]. These include, for example, in utero exposures through improvements in maternal diet, maternal adiposity, and the maternal metabolome as a result of the interventions [19, 54–56], with a possible role for persistent effects mediated via the neonatal epigenome [57, 58]. Metabolically, evidence suggests a role for maternal leptin and insulin resistance [14, 59–62]. Other metabolites may also be involved. A reduction in lipids and lipoproteins occurs in mothers following an antenatal lifestyle intervention [55], which could exert antioxidant and atheroprotective effects on both the placental endothelium and the fetus [63]. Maternal obesity may also alter epigenetic pathways associated with cardiogenesis [64, 65] and it is therefore possible that the lifestyle interventions attempt to normalise any genetic dysregulation. Studies in mice with obesity have demonstrated that maternal exercise before and during pregnancy can prevent impairments in left-ventricular function [6, 7], which appears to be due to the preservation of cardiomyocyte mitochondrial function and the reduction of sarcoplasmic reticulum calcium leakage [7]. Other animal studies suggest that maternal obesity/overnutrition during pregnancy elicits structural changes to the developing offspring hypothalamus that may, in turn, have functional consequence to offspring autonomic control and cardiovascular risk [66, 67]. These animal studies have been supported by emerging evidence from human observational studies [68, 69].

Both in utero and postnatal determinants, as well as genetic susceptibility should be considered. Whilst we cannot discount a persisting influence of lifestyle interventions on mothers, influencing the family environment and childhood cardiovascular risk [30, 54, 56], a direct in utero effect of maternal obesity becomes more likely in the context of abnormalities in fetal and neonatal cardiac structure as early as fourteen weeks’ gestation [12, 34, 70]. Furthermore, as child health measures, such as BMI, are linked with cardiovascular health [37, 48], some of the studies in this review controlled for offspring variables, attempting to account for any shared lifestyle improvements and genetic traits. Results persisted in these studies, suggesting an in utero roll for cardiac remodelling [14, 31]. However, the sample sizes were small, and controlling for offspring BMI, for example, does not capture the full picture of shared lifestyle habits and genetic traits that are linked with offspring cardiometabolic health [71, 72]. Larger follow-ups that statistically account for any shared postnatal environments and genetic traits are needed.

### Limitations and knowledge gaps

The main limitation of the included studies were the small sample sizes and large attrition rates. Although there was some evidence of improved cardiovascular development in the intervention arms, larger and statistically powered follow-ups, accounting for missingness of participants, will be needed to corroborate these results. Retention in longitudinal cohort studies is a challenge faced by many research groups [73]. As the offspring age and with the addition of further children, finding available time and childcare for study visits might be a common barrier experienced by participants. Indeed, a systematic review and meta-analysis identified that barrier-reduction strategies, such as offering childcare, assistance with transport, or home visits, appeared to be the best method to retain a greater proportion of participants [73]. Other methods such as sending participants thank you, birthday or holiday cards, and having consistent research team members showed weak evidence with improving retention rates [73]. Close attention to strategies to improve retention rates should be a focus in future follow-up studies. Studies should also utilise novel indices of cardiac remodelling, such as SSM, as these appeared to identify subtle differences in cardiac remodelling not found when standard metrics were used. With these requirements in mind, we urge groups in this field to investigate the impact of maternal obesity interventions on offspring cardiovascular remodelling.

There is evidence from human cohort studies for sex differences in cardiovascular development pre-puberty [74, 75], supported by studies of animals with obesity in pregnancy that indicate a seemingly greater adverse cardiovascular impact in male offspring [76–78]. Although some studies included participant sex as a confounding variable, only Mintjens et al. provided exploratory analyses of the effect of sex on blood pressure and vascular stiffness in response to the RCT. There was a weak trend towards a lower systolic blood pressure in female offspring from the intervention arm, but no differences in vascular stiffness or metabolic health [33]. However, the included studies in this review were likely underpowered to investigate any sexual dimorphism within the trial arms, supporting claims made by other commentators that future studies should include sex in their experimental design [78].

A meta-analysis was considered for this review, but the large heterogeneity in the method of intervention between studies (i.e. diet vs. exercise or preconception vs. antenatal) prevented statistical comparisons.

Longitudinal analysis into adolescence and adulthood are needed in future studies, as there is currently a paucity of data from late childhood onwards. Developing longitudinal trajectories and statistical methods such as causal mediation analysis will be a powerful tool to delineate the total effect of the in utero and post-natal exposures on cardiovascular outcomes in the offspring.

Most of the studies in this review were antenatal lifestyle interventions, with only one focussing on the preconception period [16, 17]. Whilst there appears to be improvements for offspring cardiovascular remodelling following antenatal interventions, there is a consensus that earlier intervention has a greater impact on pregnancy outcomes than pregnancy interventions, and may therefore have a greater influence on childhood outcomes [79, 80]. At present, it is not possible to conclude whether preconception versus antenatal interventions, or a combination of the two, have a greater impact.

## Conclusions

Well conducted RCTs can provide unique insight into the relationship between lifestyle improvements in women with obesity and cardiovascular remodelling in the child. This review provides some evidence that preconception and antenatal lifestyle interventions in women with obesity limit cardiac remodelling in the offspring. Confirmation of these findings in larger follow-up studies of older offspring, will inform public health strategies to improve the cardiovascular health of the next generation [23, 24].

### Supplementary information

Supplement material
